# Virulence Factors Found in Nasal Colonization and Infection of Methicillin-Resistant *Staphylococcus aureus* (MRSA) Isolates and Their Ability to Form a Biofilm

**DOI:** 10.3390/toxins13010014

**Published:** 2020-12-25

**Authors:** Thamiris Santana Machado, Felipe Ramos Pinheiro, Lialyz Soares Pereira Andre, Renata Freire Alves Pereira, Reginaldo Fernandes Correa, Gabriela Coutinho de Mello, Tainara Aparecida Nunes Ribeiro, Bruno Penna, Daniela Sachs, Fábio Aguiar-Alves

**Affiliations:** 1 Biotechnology and Molecular Epidemiology Laboratory, Fluminense Federal University, Niteroi, 24220-900 Rio de Jeneiro, Brazil; thamiris.santana.machado@gmail.com (T.S.M.); felipepinheiro@id.uff.br (F.R.P.); lialyz@live.com (L.S.P.A.); renatafreireapereira@gmail.com (R.F.A.P.); gabrielac.mello@hotmail.com (G.C.d.M.); 2 Pathology Postgraduate Program, Fluminense Federal University, Niteroi, 24220-900 Rio de Janeiro, Brazil; 3 Applied Microbiology and Parasitology Postgraduate Program, Fluminense Federal University, Niteroi, 24220-900 Rio de Janeiro, Brazil; 4 Santa Martha Hospital, Department of Clinical Pathology, Niterói, 24241-265 Rio de Janeiro, Brazil; correaregi@hotmail.com; 5 Microbiological Testing Laboratory Associated with Materials and Drugs of the Center for Studies, Research and Innovation in Biofunctional Materials and Biotechnology, Federal University of Itajubá, 37500-903 Itajubá, Brazil; tainara.ribeiro@unifei.edu.br (T.A.N.R.); danisachs@gmail.com (D.S.); 6 Gram-Positive Cocci Laboratory, Biomedical Institute, Fluminense Federal University, Niteroi, 24220-900 Rio de Janeiro, Brazil; bpenna@id.uff.br

**Keywords:** MRSA, resistance profile, virulence factors, biofilm, genotyping

## Abstract

Hospitalizations related to Methicillin-resistant *Staphylococcus aureus* (MRSA) are frequent, increasing mortality and health costs. In this way, this study aimed to compare the genotypic and phenotypic characteristics of MRSA isolates that colonize and infect patients seen at two hospitals in the city of Niterói—Rio de Janeiro, Brazil. A total of 147 samples collected between March 2013 and December 2015 were phenotyped and genotyped to identify the protein A (SPA) gene, the *mec* staphylococcal chromosomal cassette (SCC*mec*), *mec*A, Panton-Valentine Leucocidin (PVL), *ica*C, *ica*R, ACME, and *hla* virulence genes. The strength of biofilm formation has also been exploited. The prevalence of SCC*mec* type IV (77.1%) was observed in the colonization group; however, in the invasive infection group, SCC*mec* type II was prevalent (62.9%). The Multilocus Sequence Typing (MLST), ST5/ST30, and ST5/ST239 analyses were the most frequent clones in colonization, and invasive infection isolates, respectively. Among the isolates selected to assess the ability to form a biofilm, 51.06% were classified as strong biofilm builders. Surprisingly, we observed that isolates other than the Brazilian Epidemic Clone (BEC) have appeared in Brazilian hospitals. The virulence profile has changed among these isolates since the ACME type I and II genes were also identified in this collection.

## 1. Introduction

More than 30% of the healthy population is colonized by *Staphylococcus aureus*, making these subjects vehicles of dissemination of this pathogen [1]. For the past few years, studies conducted in Brazil have shown an increased number of colonizing Methicillin-resistant *S. aureus* (MRSA) isolates, in different age groups and with a marked antimicrobial resistance profile, pointing to the importance of monitoring this pathogen frequency and its particular characteristics, such as antimicrobial resistance patterns [2,3,4,5,6]. A high degree of similarity between nasal colonization and invasive infection isolates collected from the same patient has already been demonstrated [7,8,9], reinforcing the importance of detecting the presence of the nasal colonization and particular characteristics that could be responsible for this pathogen success and future infection development. Besides these observations, invasive nosocomial infections caused by multidrug-resistant microorganisms have been growing in recent years and account for approximately 15.5% of cases recorded worldwide [10]. Among *S. aureus* resistance mechanism*s*, the one related to beta-lactam antibiotics is highlighted [11]. It occurs due to modifications of its binding site for the altered Penicillin Binding Protein (PBP2a) encoded by the *mec*A gene, inserted in the Staphylococcal Cassette Chromosome *mec* (SCC*mec*) [12,13]. In addition to antimicrobial resistance, *S. aureus* is capable of producing a large number of virulence factors, such as toxins and enzymes, hemolysins (α, β, γ, and δ), Panton-Valentine Leucocidin (PVL), Staphylococcal Enterotoxins (SEs), exfoliative toxins A and B (ETA and ETB), toxic shock syndrome toxin (TSST-1), as well as the ACME gene, formed by the cluster *arc* and *opp3*, responsible for increasing the pathogenicity of strains by improving their fitness and conferring the ability to survive in an acidic environment [14,15,16,17,18,19].

A virulence factor, which also contributes to resistance, is used by these pathogens to form a biofilm, defined as a complex community of microorganisms surrounded by an extracellular matrix of polysaccharides adhered to each other and a surface [20]. The bacteria in biofilms can resist antimicrobials in concentrations up to 1000 times higher than those active in the same bacteria in the planktonic state [21].

An increasing number of MRSA strains are circulating in different hospitals and communities in Brazil, as well as worldwide [22,23,24,25,26]. Although the Brazilian Epidemic Clone (BEC) (ST239 SCC*mec* III) remains circulating in different hospitals and the community in our country [27,28], previous studies have shown the substitution of this clone for others, such as the New York/Japan clone (USA100/ST5/CC5/SCCmecII), community-acquired MRSA (USA300/ST8/CC8/SCCmecIV), and pediatric clone (USA800/ST5/CC5/SCCmecIV) [2,3,4,29]. This fact has evidenced the need for studies on the epidemiological, microbiological, and genetic characteristics of these microorganisms to improve and better understand the pathogen evolution, the scenario changes, and update control measures. This study compares the genotypic characteristics, besides comparing the resistance profile and ability to form biofilm among colonization and invasive MRSA isolates collected from patients attending a private and a public University Hospital in Niteroi/Rio de Janeiro, Brazil.

## 2. Results

A total of 147 samples were collected and assessed. Sixty-two samples, the majority retrieved from invasive infections (51, 83, 87%), were collected from 57 different patients from Santa Martha Hospital, and eighty-five collected from active surveillance from patients attending Santa Martha Hospital and Antonio Pedro University Hospital (HUAP). The median age of colonized patients was 47.20 ± 31.20 and 73.05 ± 19.31 for patients with invasive infections. The age range of more than 60 years old was the group with the most colonization samples (39 samples—45.88%) and invasive infection (46 samples—80.70%). Forty-six (54.12%) of the colonized patients were male, while females were prevalent among patients with invasive infection (32 patients—56.14%).

**Figure 1:**
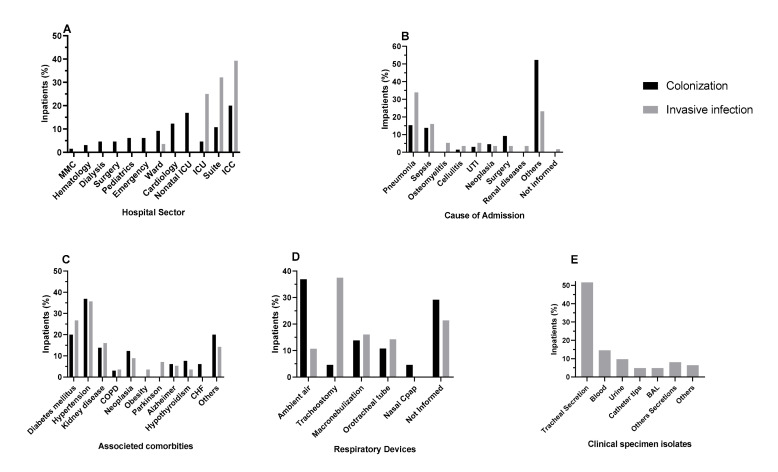
Characteristics and risk factors of colonized and invasive infection patients. (**A**) Isolates according to the hospital sectors in % values; * MMC = Male Medical Clinic; ICU = Intensive Care Unit; ICC = Intensive Care center; (**B**) cause of admission in % values for colonization and invasive infection; UTI = Urinary Tract Infection. (**C**) Associated comorbidities in % values; *COCPD = Chronic Obstructive Pulmonary Disease; CHF = Congestive Heart Failure. (**D**) Respiratory devices associated colonization and invasive infection in % values, (**E**) Clinical specimen; BAL = Bronchoalveolar lavage.

It was possible to access the complete medical records of 122 patients (65 colonized and 57 with invasive infection). Among them, the largest number of samples has been collected from the Intensive Care Unit (ICU), followed by the infection ward and Neonatal Intensive Care Unit (NICU) (Figure 1A).

Pneumonia was the main cause of admission in both groups being 10 (15.38%) in the colonization group and 19 (33.93%) in the invasive infection group, followed by sepsis (nine isolates—13.85% in colonization group and nine isolates—16.07% in the invasive infection group) (Figure 1B). Hypertension was the prevalent comorbidity in both groups, with a total of 24 (36.92%) in the colonization group and 20 (35.71%) in the invasive infection group, followed by diabetes mellitus with 13 (20.00%) and 15 (26.79%) respectively (Figure 1C). Twenty-four (36.92%) colonized patients did not have to use any respiratory devices, while tracheostomy was the respiratory procedure most used by patients presenting invasive infection (21 patients—37.50%). (Figure 1D).

Regarding the invasive infection group, most isolates (51.61%) were from tracheal secretion, nine (14.52%) from blood samples, six (9.68%) from urine samples, three (4.84%) from catheter tips, three (4.84%) from Broncho Alveolar Aspirates, five (8.06%) from diverse secretions, and four (6.45%) samples from other clinical specimens (Figure 1E).

All isolates were confirmed as *S. aureus* by MALDI-TOF analysis. The disk-diffusion method showed oxacillin and penicillin resistance for all isolates, and only two were resistant to vancomycin. The isolates from invasive infections presented a wider range of resistance to antimicrobial agents than the colonization group. The majority of 55 (88.71%) of invasive infection isolates were resistant to two or more classes of non-beta lactam antibiotics whereas, in the colonization group, only 41.18% were multi-drug resistant (MDR) (*p* ≤ 0.01) (Table 1).

Among the 85 colonization isolates, 57 (67.06%) carried the SCC*mec* IV, 27 (31.87%) the SCC*mec* II, and 1 (1.18%) the SCC*mec* III. In the 62 isolates retrieved from invasive infections, the most prevalent was the SCC*mec* type II 39 (62.90%), followed by 14 (22.6%) SCC*mec* type IV and nine (14.58%) SCC*mec* type III. None of them carried SCC*mec* type I or SCC*mec* type V (Table 2).

Eighteen Spa types were assessed among eight Sequence Types (MLST). The sequence type 5 (ST5) was prevalent among both colonization 58 (68.24%) and 46 (74.19%) invasive infection isolates. Thirteen (15.29%) colonization isolates belonged to ST30, the second most found sequence type in this group. Seven (11.29%) invasive infection isolates were typed as ST239. According to the Spa typing, t002 (ST5), 40 (47.07%) was prevalent among colonization isolates, while invasive infection samples were typed as t539 (ST5), 31(50.00%) (Table 2).

Regarding the presence of virulence factors within the colonization group, 83 samples (97.65%) carried *ica*C, 85 (100%) carried *ica*R, and 85 (100%) had *hla* genes. Similar results were observed in the invasive infection group, as 62 samples (100%) carried *ica*C and *ica*R, and 61 (98.39%) tested positive for the presence of *hla* genes. PVL genes were detected in 12 (14.12%) colonization and 4 (6.45) invasive infection isolates, without statistical significance (*p* = 0.140). PVL genes were detected in 15 isolates carrying the SCC*mec* IV and in one SCC*mec* III. All four ST8-t008 were typed as PVL positive. The ACME cluster *arc*A was mostly identified in invasive infection isolates when compared to colonization isolates (*p* < 0.01). All isolates positive for ACME cluster *opp3*AB were also positive for *arc*A. The *arc*A gene was detected in 15 isolates belonging to ST5, in the invasive infection group. In contrast, in the colonization group, this gene was found in isolates belonging to ST5 (five isolates), ST8 (one isolate), and ST239 (one isolate). Two isolates, positive for both genes, were characterized as ST8 and ST239. Only one isolate carrying SCCmec IV, ST8-t008, presented all genes tested within the colonization group. This isolate was also resistant to oxacillin, erythromycin, and penicillin (Table 2).

**Figure 2:**
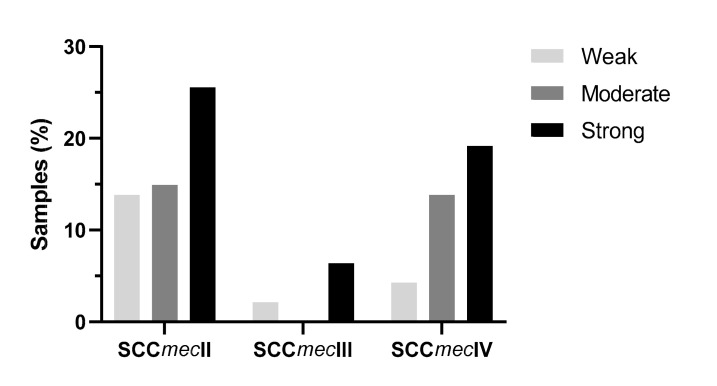
Comparative distribution between the *mec* staphylococcal chromosomal cassette (SCC*mec*) II, III, and IV isolates and their ability to form biofilms.

The classification of bacteria’s ability to form biofilm is related to the capacity of bacteria strain to adhere to a surface and form a layer, so the density of this layer was directly related to the strength of the biofilm produced. To analyze biofilm formation’s strength, 94 (63.95%) samples presented a complete genotypic identification profile, including the presence of genes related to biofilm formation, *ica*C and *ica*R, virulence genes *pvl*, *hla,* and ACME, spa typing, MLST, and SCC*mec* typing. It was possible to observe that among 51 samples of SCC*mec* II, 24 (25.53%) were considered strong, 14 (14.89%) moderate, and 13 (13.83%) weak biofilm formers. Among 8 SCC*mec* III carriers isolates, 6 (6.38%) were considered strong, and 2 (2.12%) weak biofilm formers (no moderate biofilm-forming isolate was found). Finally, among 35 carriers of SCC*mec* IV, 18 (19.15%) were strong, 13 (13.83%) moderate, and 4 (4.25%) weak biofilm formers (Figure 2).

**Figure 3:**
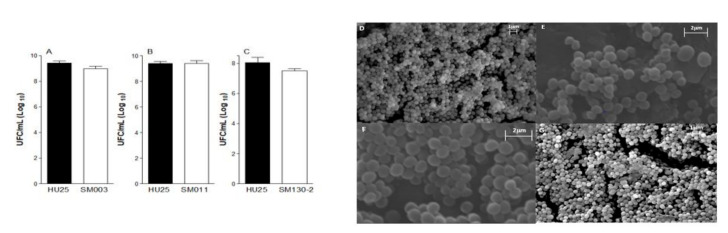
Number of colony-forming units per milliliter (CFU/mL) in *S. aureus* HU25 strong biofilm-forming strain used as the control, SM003 (**A**), SM011 (**B**), and SM130-2 (**C**) biofilms. Results are presented as the mean ± standard error mean (SEM) from 5-7 samples per group. SEM images of biofilm information of (**D**) *S. aureus* HU25, (**E**) SM003, (**F**) SM011, (**G**) SM130-2.

Of these 94 isolates, one considered strong biofilm-forming, one moderate, and one weak were selected for analysis using a scanning electron microscopy (SEM). Cell morphology was compared with that of isolate *S. aureus* HU25, a strong biofilm former (Figure 3D). The weak biofilm-forming isolate (SM003) presented low cell density, small cell clusters, and no microchannels. The bacterial cells showed normal morphology, with no changes in their cell wall (Figure 3E).

The isolate considered moderate biofilm-former (SM011—Figure 3F) presented a significant increase in cell density, and it is possible to observe different layers of cells overlapping each other. Microchannels and the absence of lesions are observed in the cell walls with a smooth and homogeneous appearance. The strong biofilm-forming isolate (SM130-2—Figure 3G) presented high cell density, where several cell clusters with several overlapping cells were observed, as well as communicating microchannels. This isolate also presented a significantly higher (*p*-value < 0.05) number of colony-forming units. In contrast, the tests performed with the samples considered moderate (SM011) and weak (SM003) biofilm-formers did not demonstrate statistical significance in the cell count when compared to the control (Figure 3A–C). However, the strains’ ability to adhere to the substrate may vary, suggesting that a good part of the colony-forming units (CFU) remained adhered to glass slide covers (GC) after washing, which may justify this difference (Figure 3A–C). However, the strength of the adhesion of the strains to the substrate may vary, suggesting that a good part of the CFU remained adhered to glass slide covers (GC), which may justify this observed difference. The weak biofilm-former tends to detach to GC in greater numbers than moderate and strong biofilm former. These observations are reflected in SEM images, where weak biofilms are less structured than moderate and strong (Figure 3A–C).

## 3. Discussion

Patients admitted to the Intensive Care Units represent 20% of the colonization group and 39.29% of the invasive infection group. This observation agrees with a report from the World Health Organization that says that the endemic burden of MRSA invasive infections is significantly higher in patients admitted to ICUs [30]. On the other hand, the same report mentions newborns as the most compromised age group. In the present study, 45.88% of colonization and 80.70% invasive infection isolates were collected from patients over 60 years old. In a 1500 sample study carried out with children from the same geographical area, MRSA isolates were responsible for colonizing 151 (10.00%) subjects [31]. A recent study showed that the prevalence of high-income children colonized by MRSA in Brazil is similar to the rates found worldwide. However, when analyzing low and middle-income or slum resident children, the prevalence is slightly higher than that found in these same countries [32]. Despite these MRSA strains having a lower virulence profile, colonization is more widespread because it poses a public health risk in cases of outbreaks since the most colonized population represents a more vulnerable portion of the population.

The use of breathing devices by colonized patients increases the chances of developing invasive infections. It is an invasive procedure and can become a vehicle of transmission through manipulation by health care professionals. Stulik et al. 2014 [33] showed the use of mechanical ventilation by patients colonized by *S. aureus* increases the risk of progression to respiratory infections such as tracheobronchitis and pneumonia associated with mechanical ventilation [33,34]. In this study, we observed 22 (33.85%) colonized patients associated with some type of respiratory device.

The most used antimicrobial agents for the treatment of MRSA invasive infections in this study were clindamycin (53/62), rifampin (19/62), sulfamethoxazole-trimethoprim (8/62), and tetracycline (5/62). It was also observed a high ciprofloxacin resistance rate (56/62) in isolates retrieved from colonized subjects. The high prevalence of multidrug-resistant strains could be explained because some of these antibiotics are the drugs of choice to treat MRSA invasive infections, leading to selective pressure from these microorganisms, justifying the antimicrobial resistance pattern found in our study [35,36].

Comparing the antimicrobial resistance burden found herein, with other studies conducted in different regions in our country, both colonization and invasive infection isolates seem to present a similar antimicrobial resistance profile. Studies conducted in the southern Brazilian region, such as Santa Catarina [37], the northern region, such as Piauí [38], Bahia [39], and Paraíba [40], and the southeastern region [2,3] with invasive infection and colonization isolates, present antimicrobial resistance profiles similar to ours. These isolates present mainly resistance to erythromycin, clindamycin, and ciprofloxacin. Although similar patterns of antimicrobial resistance are observed, the resistance frequency among the isolates in relation to tetracycline (8.06% in invasive infections and 4.7% in colonization), for example, presents a lower frequency when compared to others studies conducted in our region and other regions of Brazil [2,3,37,38,40]. Thus, showing that regional characteristics such as measures for the control and treatment of MRSA infections can influence the particularities found in circulating strains, reinforcing the need for active surveillance of circulating strains and the treatment procedure must be tailored based on the specific region of Brazil.

It seems that the pattern of high multi-drug resistance among invasive infection isolates in this study occurs by the prevalence of SCC*mec* type II (39 samples or 62.9%), normally observed as multidrug-resistant [16,41]. Colonization isolates were mainly characterized by SCC*mec* IV (57 samples or 67.1%), normally resistant only to beta-lactam antimicrobial agents [16,41]. In the present study, however, it was observed that samples carrying the SCC*mec* IV element were resistant to ciprofloxacin, erythromycin, clindamycin, and nitrofurantoin, in addition to beta-lactam.

Vieira et al. 2016 [42] observed that t002/ST5 and t318/ST30 were prevalent among HIV-positive patients and their caregivers from Rio de Janeiro and Niteroi. Similarly, in the present study, ST5 and ST30 were the most frequent, substituting the Brazilian Epidemic clone (BEC-t037-ST239).

The *pvI* genes were detected in 15 samples carrying SCC*mec*IV and one SCC*mec*III, confirming these virulence genes are not exclusive to SCC*mec*IV carriers, as generally cited [43,44,45]. On the other hand, the ACME gene formed by the *arc* and *opp*3 clusters might be responsible for the growing pathogenicity of strains circulating in Brazil. This gene is known to increase bacteria fitness, allowing for fast growth and propagating the strain [18]. Though ACME is rare among strains other than USA300, this was identified in our study in one strain USA300 and one ST239-t037-SCC*mec*III, characterized as ACME II.

No statistical significance was found indicating sample isolation site influences the ability to form biofilm and neither the presence of Panton-Valentine Leukocidin (PVL) genes (LUKF-PV and LUKS-PV). Cakir Aktas et al. 2013, Emaneini et al. 2015 and Gogoi-Tiwari et al. 2017 [46,47,48] point to a synergistic effect of these genes in increased virulence, but not directly on the strength of biofilm formation. However, when investigating the presence of the ACME gene clusters, a tendency to form strong biofilms can be observed with statistical relevance [49]. This information was corroborated by this study, since the colonization sample SM130-2, the only carrier of the two clusters of the ACME genes (*arc*A and *opp*3AB), showed an increased ability to form biofilm when compared to the other strains.

We identified 22 ST5 samples carrying only the *arc*A cluster and were characterized as having a truncated form of ACME I, called ACME II, identified by other studies [18,50,51,52]. Identifying many ST5 isolates causing invasive infection by carrying the ACME gene is of great concern. These strains are multidrug-resistant, and the presence of this gene might increase their pathogenicity, allowing them to grow faster [50,52,53].

Only one sample was positive for all of the genes tested in this study. It was typed as USA300 (ST8/t008), an endemic clone in the United States [54]. Although this strain is resistant only to erythromycin and beta-lactam antibiotics, the ACME gene’s presence increases its pathogenicity. It may increase the risk of invasive infections, both in the community and hospital environments.

Analyzing the strength of biofilm formation, non-biofilm-forming strains were not observed, which could be explained by the presence of different genes regulating biofilm formation, commonly carried by these strains, such as poly-β(1-6)-N-acetylglucosamine/polysaccharide intercellular adhesin (PNAG/PIA). On the other hand, SCC*mec* II carrier strains have a greater tendency to produce strong biofilms, as observed by Pozzi et al. 2012 [55].

We observed a higher cell density in strains considered moderate biofilm-formers through image analysis compared to those classified as strong. This may suggest that these analyses are important for developing strategies to fight infections, but not following clinical damage they may cause.

## 4. Conclusions

Invasive infection isolates have a higher resistance to the antimicrobial agents tested when compared to nasal colonization samples. Several different SCC*mec* types among nasal colonization and invasive infection samples were also observed. New clones seem to be substituting the Brazilian Epidemic Clone in hospital and community environments. The PVL genes were not found only in SCC*mec*IV isolates, but also in strains carrying SCC*mec*III. ACME type I and the truncated form of ACME type II were identified for the first time in Brazil. Invasive infection isolates presented more pathogenicity-related genes when compared to nasal colonization samples.

A total of 94 (51.06%) isolates were classified as strong biofilm formers. No relationship has been observed between the presence of PVL genes and biofilm formation. However, ACME gene carriers were all in the group of strong biofilm formers. The relation of the ACME genes and staphylococci biofilms remains unclear and new studies should be carried out to investigate this. *S. aureus* carrying SCC*mec*II also tended to form stronger biofilms. The ability to form a biofilm, as a resistance factor, draws our attention because it makes infections more difficult to treat. Strong biofilm-forming strains, which are also MDR, associate the difficulty of eradicating planktonic cells and also the sessile ones.

There was no relationship between the sample’s anatomical site of origin with the strain’s ability to form a biofilm, nor significant differences between the ability to form biofilm among samples of invasive infection or colonization.

## 5. Materials and Methods

### 5.1. Data Collection

This cross-sectional cohort study covers colonizing and invasive or/and non-invasive isolates from Santa Martha Hospital and Antonio Pedro University Hospital, collected from March 2013 to December 2015. Patient data, including gender, age, hospital ward, cause of admission, comorbidities, type of sample, and use of respiratory devices, were collected from patient medical records.

### 5.2. Samples Acquisition and Characterization

Samples were collected using a dry swab in infected sites and stored in the Stuart transport medium (Labor import-Brazil, Sāo Paulo- Brazil). Swabs were also collected from both nostrils of inpatients for MRSA colonization surveillance. Characterization, phenotypic tests, and species confirmation was performed by the VITEK^®^ system (bioMérieux Inc., Lyon, France) and Matrix-Assisted Laser Desorption Ionization—Time of flight (MALDI-TOF) (Bruker Billerica, MA, USA), According to Sogawa et al., 2017 [56], with slight modifications.

### 5.3. Antimicrobial Susceptibility Test

The antimicrobial profile was confirmed by antimicrobial susceptibility test using the disk-diffusion method Kirby-Bauer, as recommended by the Clinical and Laboratory Standards Institute [57], to confirm the resistance profile. The antimicrobial agents tested were: ciprofloxacin (5 μg), chloramphenicol (30 μg), clindamycin (2 μg), erythromycin (15 μg), nitrofurantoin (300 μg), gentamicin (10 μg), cefoxitin (30 μg), oxacillin (1 μg), rifampin ( 5 μg), sulfamethoxazole-trimethoprim (23.75 μg/1.25 μg), tetracycline (30 μg), vancomycin (30 μg), and penicillin (10 I.U..) and *S. aureus* HU25 as a positive control [31].

### 5.4. Genotyping

DNA extraction from Methicillin-Resistant *S. aureus* (MRSA) was performed using the QIAamp^®^ DNA mini kit (QIAGEN, Hilden, Germany), according to the manufacturer’s instructions. SCC*mec* typing, Spa typing, and Multilocus Sequence Typing (MLST) were performed in all isolates as described previously [31,43,58,59,60]. To detect β-lactam resistance (*mec*A), virulence factors [PVL, α-hemolysin, and ACME clusters (*arc*A and *opp*3AB)] and biofilm-related (*ica*C and *ica*R) genes, samples were tested by PCR, as previously described [18,43,61,62]. The presence of *ica*C e *ica*R was confirmed by PCR, using the primers icaC179F- TGTCACAGTTACTGACAACCT and icaC986R- CGCAATATCATGCCGACACC, and icaR40F-TCAGAGAAGGGGTATGACGGT and icaR496R- CCACTGCTCCAAATTTTTGCG, respectively. The control strains used for the genotyping reactions are listed in Table S1 (Supplementary Material). The PCR was performed as follows: Buffer 5 × 1 (Promega, WI, USA), 25 mM MgCl2 (Promega Biosciences Inc., Madison, WI, USA), 2 mM dNTP (Promega, WI, USA), 10 µM of each primer, Ampli Taq Gold DNA polymerase (Promega, WI, USA), DNA and Milli-Q water with a total volume of 25 µL. DNA was amplified in a Nexus Gradient Mastercycler (Eppendorf, Hamburg, Germany), with an initial denaturation step (94 °C for 5 min), 30 cycles of denaturation (95 °C for 30 s), annealing (55 °C for 30 s) and extension (72 °C, 1 min), and a final extension step (72 °C for 7 min). Amplicons were analyzed on 1% agarose gel in TBE 0.5×. (Controls were shown on ST1).

### 5.5. Evaluation of Biofilm Strength

Isolated colonies were inoculated into 5 mL of sterile NaCl 0,9% solution to match a 0.5 McFarland scale (~1.5 × 10^8^ CFU) and incubated at 37 °C for 24 h. A total of 200 µL of the sample diluted 1:100 on Brain Heart Infusion (BHI) supplemented with glucose 1% has been transferred to a 96-well polystyrene NUNC plate (Thermo Fisher Scientific, Waltham, MA, USA) in triplicates, following another 37 °C/24 h incubation. Plates were then washed with distilled water following fixation by heat for 1 h at 60 °C, stained with crystal violet 0.1% for 15 min at Room Temperature, washed with running tap water, air-dried, and finally re-solubilized with 150 µL of ethanol 99% P.A. for 30 min. The optical density (OD) was measured at 570 nm using a microtiter plate reader with a wet plate [63]. Biofilms were classified as comparing the cut-off (the mean optical density of the blank plus three times the standard deviation (Cut Off (CO) = Optical Density OD × Blank (BK) + 3 × Standard Deviation (SD) and values of samples OD. Values below the cut-off were considered to belong to a group of non-biofilm-forming isolates. Strains with absorbance values up to twice the cut-off were considered weak biofilm formers. In contrast, those presenting two and four times the cut-off were defined as moderate biofilm formers, and four times the cut-off were considered to be related to a strong biofilm-forming group [63].

### 5.6. Biofilm Scanning Electron Microscopy (SEM) and CFU Counting

The inoculum was adjusted to 0.5 McFarland standard. A total of 36 sterilized disks of glass slide covers (GC) were used to measure and visualize the biofilm growth. After sterilization, the GCs were randomly assigned to four different groups: *S. aureus* HU25 (a strong biofilm-forming strain used as the control), SM003, SM011, and SM130-2 (*n* = 9 GC for each group). GCs were placed in a 24-well polystyrene plate (Techno Plastic Products AG-TPP, Trasadingen, Switzerland) with 1 mL of BHI-g broth and 0.1 mL of the inoculum. The plates were incubated for 24 h at 37 °C, and then, from each group, two (2) GCs were assigned to perform SEM, and the remaining seven (7) GCs were selected for colony-forming units (CFU) counting. For SEM assessment, each GC went through a sequence of preparation steps. First, they were aseptically washed with 3 mL of sterile 0.9% saline, fixed in 2.5% glutaraldehyde for 1 h, and dehydrated in several washes with different concentrations of ethanol (10, 25, 50, 75, and 90% for 20 min and 99.5% for 1 h). Samples were dried overnight in a bacteriological incubator at 37 °C and then coated with gold in a low-pressure atmosphere with ion sputter. The biofilms’ topographies were visualized and photographed using a scanning electron microscope (Carl Zeiss EVO^®^ MA15 or Shimadzu, SS-550 SUPERSACAN, Kyoto, Japan). To count colony-forming units, a total of seven (7) GC samples were aseptically washed with 3 mL of sterile 0.9% saline, placed in tubes with 10 mL of sterile 0.9% saline, and sonicated (USC -1400A, Unique-Indaiatuba, Sao Paulo, Brazil) for 10 min to disperse the biofilms from glass slide covers. The original bacterial suspension recovered was diluted from 10-2 to 10-8 by adding sterile 0.9% saline. Aliquots of 10 µL were seeded on BHI agar plates and incubated at 37 °C for 24 h. The number of colony-forming units per milliliter (CFU/mL) was determined, and the results were presented in a log (log10) chart [64].

### 5.7. Statistical Analysis

Bivariate analysis was performed using the Chi-square test (χ^2^), and values of *p* ≤ 0.05 were considered statistically significant. For the biofilm strength classification, analyzes of the standard deviation of the mean per sample and Chi-square test and Fisher’s exact test were used to define the relationship between the strength of biofilm formation and virulence factors among all tested isolates. The *t*-test was also performed to assess biofilm formation’s strength based on the detection of virulence genes.

### 5.8. Ethical Statement

The study was conducted following the Declaration of Helsinki of 1975, and the protocol was approved by the Ethics Committee in Human Research at Federal Fluminense University (108.042) under the number: 01097412.6.0000.5243. The approval date is 26 September 2012. As data analysis was retrospective, and after patient discharge, no signed consent, apart from the general one for hospitalization, was obtained.

## Figures and Tables

**Table 1:** Resistance profile among nasal colonization and invasive infection samples.

Antibiotics	Colonization	Invasive Infection	*p*
	(*n* = 85)	(*n* = 62)	
Ciprofloxacin	26 (30.59)	56 (90.32)	<0.001
Clindamycin	29 (34.12)	53 (85.48)	<0.001
Chloramphenicol	6 (7.06)	5 (8.06)	0.818
Erythromycin	43 (50.59)	54 (87.10)	<0.001
Gentamicin	4 (4.71)	11 (17.74)	<0.01
Nitrofurantoin	4 (4.71)	0	0.083
Rifampin	9 (10.59)	19 (30.65)	<0.01
Trimethoprim/Sulfamethoxazole	3 (3.53)	8 (12.90)	<0.05
Tetracycline	4 (4.71)	5 (8.06)	0.401
Vancomycin	2 (2.35)	0	0.224

**Table 2:** Genetic profile of nasal colonization and invasive infection isolates.

Sequence Type	Spa type	*mec*A	SCC*mec*	Genes	Colonization	Invasive Infection
					(*n* = 85)	(*n* = 62)
ST1	t127	+	IV	*hla*, *ica*C, *ica*R	3	2
	t922	+	IV	*hla*, *icaC*, *icaR*	0	1
ST5	t002	+	II	*hla*, *ica*C, *ica*R	8	9
		+	IV	*hla*, *ica*C, *ica*R	30	4
		+	IV	*hla*, *ica*R	1	0
		+	IV	*hla*, *ica*C, *ica*R, *pvl*	0	1
		+	IV	*hla*, *ica*C, *ica*R, *arc*A	1	0
	t045	+	IV	*hla*, *ica*C, *ica*R	1	0
		+	IV	*hla*, *ica*C, *ica*R, *pvl*	1	0
	t067	+	II	*hla*, *ica*C, *ica*R	4	0
	t306	+	IV	*hla*, *ica*C, *ica*R	0	1
	t311	+	IV	*hla*, *ica*C, *ica*R	1	0
	t539	+	II	*hla*, *ica*C, *ica*R	5	14
		+	II	*icaC*, *icaR*	0	1
		+	II	*hla*, *ica*C, *ica*R, *arc*A	5	13
		+	III	*hla*, *ica*C, *ica*R	0	2
		+	IV	*hla*, *ica*C, *ica*R	1	0
		+	IV	*hla*, *ica*C, *ica*R, *arc*A	0	1
ST8	t008	+	IV	*hla*, *ica*C, *ica*R, *pvl*, *arc*A, *opp*3AB	1	0
		+	IV	*hla*, *ica*C, *ica*R, *pvl*	2	1
ST30	t021	+	IV	*hla*, *ica*C, *ica*R	2	1
		+	IV	*hla*, *ica*C, *ica*R, *pvl*	1	1
	t318	+	IV	*hla*, *ica*C, *ica*R	4	0
		+	IV	*hla*, *ica*C, *ica*R, *pvl*	3	0
		+	IV	*hla*, *ica*R, *pvl*	1	0
	t433	+	IV	*hla*, *ica*C, *ica*R	0	1
		+	IV	*hla*, *ica*C, *ica*R, *pvl*	0	1
	t964	+	IV	*hla*, *ica*C, *ica*R	1	0
		+	IV	*hla*, *ica*C, *ica*R*,pvl*	1	0
ST45	t004	+	II	*hla*, *ica*C, *ica*R	0	1
	t266	+	II	*hla*, *ica*C, *ica*R	4	0
ST97	t359	+	IV	*hla*, *ica*C, *ica*R	1	0
		+	IV	*hla*, *ica*C, *ica*R*,pvl*	1	-
ST239	t037	+	III	*hla*, *ica*C, *ica*R	0	6
		+	III	*hla, icaC, icaR, pvl*	0	1
		+	III	*hla*, *ica*C, *ica*R, *arc*A, *opp*3AB	1	0
ST950	t895	+	II	*hla*, *ica*C, *ica*R	1	0
Total					85	62

+: positive for *mec*A gene.

## Supplementary Materials

The following are available online at https://www.mdpi.com/2072-6651/13/1/14/s1, Table S1: Control strains.
