# Supplementary material for: Virulence Factors Found in Nasal Colonization and Infection of Methicillin-Resistant Staphylococcus aureus (MRSA) Isolates and Their Ability to Form a Biofilm
Source: Toxins (Basel). 2020 Dec 25;13(1):14. doi: 10.3390/toxins13010014 (PMC7823648; doi:10.3390/toxins13010014)
Supplement: Supplementary file 1 [file toxins-13-00014-s001.pdf]

Supplementary Materials: Virulence Factors Found in Nasal Colonization and Infection of Methicillin-Resistant *Staphylococcus aureus* (MRSA) Isolates and Their Ability to Form a Biofilm

Thamiris Santana Machado, Felipe Ramos Pinheiro, Lialyz Soares Pereira Andre, Renata Freire Alves Pereira, Reginaldo Fernandes Correa, Gabriela Coutinho de Mello, Tainara Aparecida Nunes Ribeiro, Bruno Penna, Daniela Sachs and Fábio Aguiar-Alves

Table S1. Control strains.

Gene	Positive Control	Negative Control	Observations
***mec*A**	HU25	MSSA pvl+	HU25 see [1]
pvl	WB45	HU25	WB45 see [2]
**SCC*mec* I**		Distilled water	
**SCC*mec* II**	USA 100	Distilled water	
**SCC*mec* III**	HU25	Distilled water	
**SCC*mec* IV**	WB45	Distilled water	
**SCC*mec* V**		Distilled water	
**ACME**	FPR	USA 100	FPR is USA300 ST8, *pvl* +, ACME+ SCC*mec*IV
hla	FPR	E. coli ATCC 11775	
***ica*C**	HU25	E. coli ATCC 11775	
***ica*R**	HU25	E. coli ATCC 11775	

References:

1. Teixeira, L.A.; Resende, C.A.; Ormonde, L.R.; Rosenbaum, R.; Figueiredo, A.M.; de Lencastre, H.; Tomasz, A. Geographic spread of epidemic multiresistant Staphylococcus aureus clone in Brazil. *J. Clin. Microbiol.* **1995**, *33*, 2400–2404, doi:10.1128/JCM.33.9.2400-2404.1995.
2. Ribeiro, A.; Dias, C.; Silva-Carvalho, M.C.; Berquó, L.; Ferreira, F.A.; Santos, R.N.S.; Ferreira-Carvalho, B.T.; Figueiredo, A.M. First Report of Infection with Community-Acquired Methicillin-Resistant Staphylococcus aureus in South America. *J. Clin. Microbiol.* **2005**, *43*, 1985–1988, doi:10.1128/JCM.43.4.1985-1988.2005.
